# The effect of synthetic pyrethroids on the attachment and host-feeding behaviour in *Dermacentor reticulatus* females (Ixodida: Amblyommidae)

**DOI:** 10.1186/s13071-015-0977-0

**Published:** 2015-07-11

**Authors:** Alicja Buczek, Patrycja Lachowska-Kotowska, Katarzyna Bartosik

**Affiliations:** Department of Biology and Parasitology, Medical University, Radziwiłłowska11 St.,, 20-080 Lublin, Poland

**Keywords:** *Dermacentor reticulatus*, Permethrin, Deltamethrin, Attachment behaviour, Host-feeding behaviour

## Abstract

**Background:**

The high competence of *D. reticulatus* in transmission of tick-borne pathogens prompts investigations of the effect of chemicals used as repellents and acaricides on the behaviour of the tick on the host. Therefore, this paper presents the effect of permethrin and deltamethrin on the attachment and feeding in this tick species.

**Findings:**

Attachment to rabbit skin of *D. reticulatus* females sprayed with pyrethroids and the effect of different doses thereof on feeding were assessed at a temperature of 20 ± 3 °C and 50 % humidity.

The dynamics of attachment of *D. reticulatus* females varied in a dose-dependent manner after the application of both pyrethroids. Within the first 0.5 h of the experiments, there was an over six-fold and over twelve-fold increase in the number of females attached to host skin after application of permethrin concentrations of 0.3906–0.7812 μg and 1.5625–3.1250 μg/1 specimen, respectively. In the case of deltamethrin, females treated with the dose of 0.0390 μg of the compound were able to attach to host skin only 4 hours after the infestation.

The toxic activity of both pyrethroids increased the duration of the feeding period and decreased the body weight of engorged females and the feeding efficiency index.

**Conclusions:**

The accelerated attachment of *D. reticulatus* females caused by sublethal permethrin doses and delayed or inhibited attachment caused by deltamethrin suggest a necessity of careful selection of the type and dose of pyrethroids to protect hosts from tick attacks.

## Background

The most effective method for reduction of the threat posed by ticks to human [1] and animal health is to use repellents and acaricides. However, chemicals can produce adverse effects, e.g. behavioural changes and toxicoses in animals [2, 3] as well as disturbances in the function of various systems in humans [4]. Moreover, long-term use and/or application of inadequate doses of the compounds may result in development of resistance of ticks to these chemicals [5, 6].

Among the chemicals applied in tick control, synthetic pyrethroids are widely used as repellents for protection of humans [7–9] and animals [10–12] and as tick control agents [13–18].

In order to achieve higher effectiveness of pyrethroids in tick control and to reduce the disease-related effects of tick parasitism, detailed knowledge about their effect on different species of ticks in the parasitic phase of their life cycle is particularly important. Therefore, this paper describes for the first time the effect of sublethal doses of permethrin and deltamethrin on the attachment and feeding behaviour of females of the meadow tick *Dermacentor reticulatus* (Fabricius), a common species in large areas of the Palearctic.

## Methods

### Acaricides

Hungry females were treated with formulations of two pyrethroids: permethrin (Coopex 25WP) and deltamethrin (K-Othrine 2,5 flow). Each hungry female received a single application of 10 μl of one of the tested pyrethroid solutions (Table 1).

**Table 1:** Quantity of active substance in 10 μl of a permethrin and deltamethrin solutions applied as a single dose (in μg)

Concentration of the solution	Permethrin (μg)	Deltamethrin (μg)
(%)		
0.0156	0.3906	0.0390
0.0312	0.7812	0.0781
0.0625	1.5625	0.1562
0.1250	3.1250	0.3125
0.2500	6.2500	0.6250

### Ticks

The *D. reticulatus* adults examined in this experiment were collected in the Lublin macroregion (51°15'N, 22°36'E) between September and October. Before acaricide tests, adult *D. reticulatus* ticks were kept in glass rearing chambers at room temperature of ca. 20 °C and 90 % humidity. Only morphologically intact ticks were used.

### Acaricide testing procedure

Each female was weighed with an accuracy of 0.0001 g using an analytical digital balance; next, the pyrethroid solutions were applied with a micropipette onto its dorsal side. The next day, the females and untreated males were placed on rabbits’ skin.

Each pyrethroid solution was tested on 3 New Zealand albino rabbits, each of which was infested by 10 females and 5 males. The course of tick attachment and feeding on the host was observed at a temperature of 20 ± 3 °C and 50 % humidity.

The course of attachment to the host skin in each experimental group of *D. reticulatus* females and in the control group was assessed at 0.5-h intervals for 7 h, i.e. until the attachment of the last female. After the ticks began feeding, the experimental animals were viewed once a day at the same time and detached females were collected. Next, the females were weighed using an analytical balance and transferred to rearing chambers.

The experiments in the control group were conducted analogously to the pyrethroid tests, but the females were treated with 10 μl of distilled water instead of the solution of the chemical substance.

In all the experimental groups, attachment of *D. reticulatus* females to the host and the course of the parasitic phase were assessed based on several parameters (Tables 2, 3 and 4).

**Table 2:** Attachment of *D. reticulatus* females (%) under the influence of pyrethroids studied and in control group at temperature 20 ± 3 °C and 50 ± 3° % RH

Pyrethroid	Concentration (%)	Time (h)													
		0.5	1	1.5	2	2.5	3	3.5	4	4.5	5	5.5	6	6.5	7
Permethrin	0.015625	25	50	50	50	50	50	100	100	100	100	100	100	100	100
	0.03125	25	25	25	25	50	50	75	75	100	100	100	100	100	100
	0.0625	67	100	100	100	100	100	100	100	100	100	100	100	100	100
	0.125	50	50	50	100	100	100	100	100	100	100	100	100	100	100
Deltamethrin	0.015625	0	0	0	0	0	0	0	100	100	100	100	100	100	100
Control		4.0	24.0	36.0	48.0	56.0	68.0	80.0	88.0	92.0	96.0	96.0	96.0	96.0	100.0

**Table 3:** Parameters of parasitic phase in *Dermacentor reticulatus* females infesting the rabbit host, after application of permethrin solutions on unfed females at temperature 20 ± 3 °C and 50 ± 3° % RH

Parameter	Statistics	Concentration of the solution (%)				Control	H Test
		0.0156	0.0312	0.0625	0.1250		
Feeding period (days)	Min	12	7	9	9	6	6.2085
	Max	17	17	13	13	16	
	M	14	12	10	11	9	
	SD	2.9	4.1	2.3	2.8	3,4	p = 0.1841
	U Test	10.0	24.0	18.5	11.5	x	
	p	0.0605	0.1821	0.2564	0.2999	x	
Female engorged weight (g)	Min	0.1719	0.1294	0.1639	0.1294	0,1468	9.0366
	Max	0.2772	0.3469	0.3185	0.3112	0,4637	
	M	0.2346	0.2473	0.2380	0.2203	0,3329	
	SD	0.0554	0.0898	0.0775	0.1286	0,0811	p = 0.0602
	U Test	9.0	18.0	11.0	8.0	x	
	p	0.0495	0.0752	0.0735	0.1560	x	
Feeding efficiency index *(g/24 h)	Min	0.0143	0.0108	0.0126	0.0100	0,0092	9.3192
	Max	0.0212	0.0496	0.0354	0.0346	0,0740	
	M	0.0173	0.0243	0.0246	0.0223	0,0421	
	SD	0.0035	0.0175	0.0114	0.0174	0,0181	p = 0.0536
	U Test	7.0	20.0	12.0	7.0	x	
	p	0.0324	0.1029	0.0887	0.1266	x	

*average increase in body mass (in g) per 24 h, M – mean; SD – standard deviation, at permethrin concentrations 0.25 % i 0.5 % no *D. reticulatus* female was able to complete feeding

**Table 4:** Parameters of parasitic phase in *Dermacentor reticulatus* females infesting the rabbit host,after application of deltamethrin solutions on unfed females at temperature 20 ± 3 °C and 50 ± 3°% RH

Parameter	Statistics	Concentrationof the solution(%)	Control
		0.0156	
Feeding period (days)	Min	11	6
	Max	12	16
	M	12	9
	SD	0.7	3.4
	UTest	10.0	x
	p	0.2300	x
Female engorged weight (g)	Min	0.2218	0.1468
	Max	0.2497	0.4637
	M	0.2358	0.3329
	SD	0.0197	0.0811
	UTest	4.0	x
	p	0.0636	x
Feeding efficiency index* (g/24 h)	Min	0.0202	0.0092
	Max	0.0208	0.0740
	M	0.0205	0.0421
	SD	0.0005	0.0181
	UTest	8.0	x
	p	0.1560	x

*Average increase in body mass (in g) per 24 h,M – mean; SD – standard deviation, at deltamethrin concentrations 0.03125 %; 0.0625 %; 0.125 %; 0.25 % i 0.5 % no *D. reticulatus* female was able to complete feeding

### Statistical analysis

The U Mann -Whitney test was employed to check whether there were significant differences in the values of the parameters between the control and experimental groups. With the use of the H Kruskal-Wallis test, we verified the hypothesis of equality of the parameters for the different concentrations of the acaricides applied. In both tests, a significant statistical difference was established at p ≤ 0.05 and a highly significant difference at p ≤ 0.01.

### Ethical approval

The study was performed with the full approval of Commission for Animal Experiments.

## Findings

**Fig. 1:**
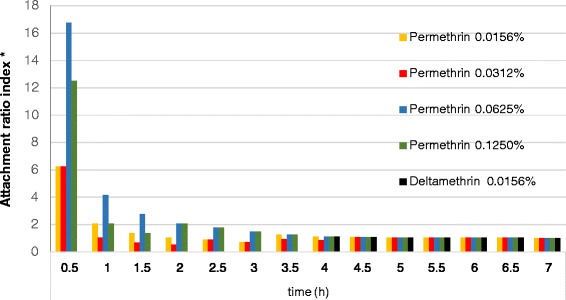
Attachment ratio index^*^ of *Dermacentor reticulatus* females under the influence of pyrethroids studied; the study was performed at temperature 20 ± 3 °C and 50 ± 3 % RH,*the ratio of the average percentage of attached females in the experimental group to the average percentage of attached females in the control group at a specific time of the experiment

Permethrin and deltamethrin applied at sublethal doses altered the host-feeding behaviour of *D. reticulatus* ticks. Unengorged females treated with permethrin attached themselves to the host earlier than the females from the control group (Table 2). Within the first 0.5 h of the experiment, an over six-fold greater number of females treated with 0.390625 to 0.78125 μg of permethrin were found attached to rabbits’ skin than in the control group. Even greater differences between the tested and control groups were observed at the application of the higher concentrations of permethrin, which was confirmed by the values of the attachment index ratio (Fig. 1). Depending on the dose, deltamethrin delayed or completely prevented the attachment of tick females to rabbits’ skin (Table 2). Unengorged, deltamethrin-treated *D. reticulatus* females attached themselves to the host and started feeding 4 hours after host infestation only when the 0.0390 μg dose was applied (Table 2). The attachment rate index at this point of the experiment was only 1.13 (Fig. 1).

The treatment of *D. reticulatus* females with both pyrethroids resulted in prolongation of the feeding period, but in comparison with the control group, these differences were not statistically significant (Table 3 and 4). After a longer feeding period, females treated with the 0.3906 μg permethrin dose exhibited a statistically significantly decrease in body weight and their feeding efficiency decreased compared with the control (Table 3). The feeding efficiency declined, reaching the threshold of statistically significant differences in the female group treated with the different concentrations of permethrin (Table 3).

After the application of the tested deltamethrin dose, the values of the feeding parameters decreased, but did not exhibit statistically significant differences, compared with the control (Table 4).

## Discussion

Our research, carried out on unengorged *D. reticulatus* specimens for the first time, has shown that sublethal doses of permethrin increase the activity of hungry females of this species, which results in higher dynamics of attachment to host skin. This change in tick behaviour induced by application of permethrin may be associated with its toxic effect on synganglion cells in ticks, which has been documented by other authors [19–21].

Morphological changes and, consequently, impaired secretion of neurotransmitters can cause disturbances in conduction of nerve impulses not only in the synganglion cells but also in other organs controlled by the nervous system [22, 23]. The effects of the toxic effects of permethrin include morphophysiological changes in the reproductive organs, [21] which reduce the reproductive performance in tick females [15, 16], and changes in tick salivary glands [20, 24, 25] resulting in impairment of their secretory and osmoregulatory function.

The differences in the behaviour of hungry *D. reticulatus* females on the host induced by permethrin and deltamethrin are related to the different toxicity of both these substances and the different mechanism of action of these pyrethroids. In our previous studies, we reported that deltamethrin applied to hungry and engorged *D. reticulatus* females produced stronger toxic effects than permethrin [16].

In these investigations, the toxic effect of pyrethroids on *D. reticulatus* females during the parasitic phase was manifested by changes in the analysed parameters. The reduced feeding efficiency index indicates changes in the dynamics and/or impaired secretion of saliva components, which play an important role in attachment of ticks to the host, digestion of host tissues, inhibition of host immune response, and transmission of some pathogens.

As shown in our research, changes in tick attachment and feeding induced by the use of pyrethroids may result in adverse interactions between ticks and the host and have a detrimental effect on the host. This issue, however, needs elucidation.
